# Rapid diagnostic tests for molecular surveillance of *Plasmodium falciparum* malaria -assessment of DNA extraction methods and field applicability

**DOI:** 10.1186/1475-2875-12-106

**Published:** 2013-03-19

**Authors:** Ulrika Morris, Berit Aydin-Schmidt, Delér Shakely, Andreas Mårtensson, Louise Jörnhagen, Abdullah S Ali, Mwinyi I Msellem, Max Petzold, José P Gil, Pedro Ferreira, Anders Björkman

**Affiliations:** 1 Malaria Research, Department of Medicine Solna, Karolinska Institutet, Stockholm, Sweden; 2 Department of Medicine, Kungälv Hospital, Kungälv, Sweden; 3 Department of Public Health Sciences, Division of Global Health (IHCAR), Karolinska Institutet, Stockholm, Sweden; 4 Zanzibar Malaria Control Programme (ZMCP), Ministry of Health, Zanzibar, Tanzania; 5 Centre for Applied Biostatistics, Sahlgrenska Academy, University of Gothenburg, Gothenburg, Sweden; 6 Drug Resistance and Pharmacogenetics Group, Institute of Biotechnology and Bioengineering, Centre of Molecular and Structural Biomedicine, University of Algarve, Faro, Portugal; 7 Laboratory of Molecular Anthropology and Health, Department of Anthropology, Binghamton University, Binghamton, NY, USA; 8 Department of Physiology and Pharmacology, Drug Resistance Unit, Section of Pharmacogenetics, Karolinska Institutet, Stockholm, Sweden; 9 Department of Protozoology, Institute of Tropical Medicine, Nagasaki University, Nagasaki, Japan

**Keywords:** *Plasmodium falciparum*, Malaria, Rapid diagnostic test, DNA extraction, Molecular surveillance

## Abstract

**Background:**

The need for new malaria surveillance tools and strategies is critical, given improved global malaria control and regional elimination efforts. High quality *Plasmodium falciparum* DNA can reliably be extracted from malaria rapid diagnostic tests (RDTs). Together with highly sensitive molecular assays, wide scale collection of used RDTs may serve as a modern tool for improved malaria case detection and drug resistance surveillance. However, comparative studies of DNA extraction efficiency from RDTs and the field applicability are lacking. The aim of this study was to compare and evaluate different methods of DNA extraction from RDTs and to test the field applicability for the purpose of molecular epidemiological investigations.

**Methods:**

DNA was extracted from two RDT devices (Paracheck-Pf® and SD Bioline Malaria Pf/Pan®), seeded *in vitro* with 10-fold dilutions of cultured 3D7 *P. falciparum* parasites diluted in malaria negative whole blood. The level of *P. falciparum* detection was determined for each extraction method and RDT device with multiple nested-PCR and real-time PCR assays. The field applicability was tested on 855 paired RDT (Paracheck-Pf) and filter paper (Whatman® 3MM) blood samples (734 RDT negative and 121 RDT positive samples) collected from febrile patients in Zanzibar 2010. RDT positive samples were genotyped at four key single nucleotide polymorphisms (SNPs) in *pfmdr1* and *pfcrt* as well as for *pfmdr1* copy number, all associated with anti-malarial drug resistance.

**Results:**

The *P. falciparum* DNA detection limit varied with RDT device and extraction method. Chelex-100 extraction performed best for all extraction matrixes. There was no statistically significant difference in PCR detection rates in DNA extracted from RDTs and filter paper field samples. Similarly there were no significant differences in the PCR success rates and genotyping outcomes for the respective SNPs in the 121 RDT positive samples.

**Conclusions:**

The results support RDTs as a valuable source of parasite DNA and provide evidence for RDT-DNA extraction for improved malaria case detection, molecular drug resistance surveillance, and RDT quality control.

## Background

The World Health Organization has recommended the use of malaria rapid diagnostic tests (RDTs) for prompt and accurate parasitological confirmation of *Plasmodium falciparum* malaria in settings where microscopy services are not available. However, the ability to detect individuals with asymptomatic low density parasitaemia, i.e., below detection limit of both RDTs and microscopy (~100 parasites/μL blood), in low endemic settings has been increasingly acknowledged as a challenge to achieve malaria elimination [1]. In this context there is a need for novel sensitive molecular tools and strategies for improved malaria case detection. Furthermore, molecular tools for monitoring the selection of genotypes associated with anti-malarial drug resistance are critical since they may provide an early warning system of development and spread of tolerance/resistance to artemisinin-based combination therapy (ACT) before clinical treatment failures are apparent.

The possibility of recovering parasite DNA from RDTs was first shown by Veron *et al.*[2]. Thereafter, two additional methods of DNA extraction from RDTs have been published [3,4] suggesting that RDTs are a reliable source for parasite DNA preservation. This provides an opportunity for improved molecular surveillance and RDT quality control. However, and importantly, comparative studies on the efficiency of the published DNA extraction methods from RDTs are lacking. This is critical since there is evidence that the efficiency of DNA extraction may be highly influenced by choice of extraction matrix and method [3-6]. Furthermore, comprehensive studies are needed to investigate whether wide-scale collection of RDTs can provide the basis for modern molecular surveillance of malaria, including both improved malaria case detection and anti-malarial drug resistance genotyping.

The aims of this study were, therefore, to evaluate two published DNA extraction methods, in comparison with a previously unpublished, high-throughput method (Ferreira P E, unpublished data), and to assess the field applicability of RDT-DNA extraction for molecular surveillance, in comparison to DNA extraction from filter paper.

## Methods

### RDT-DNA extraction methods

Three DNA extraction methods from RDTs were evaluated: 1) A simple elution method [3]; 2) Chelex-100 extraction [4]; and, 3) a previously unpublished method following a modified version of the protocol “isolation of DNA from fresh or frozen whole blood” employing an ABI PRISM 6100 Nucleic Acid PrepStation™ and NucPrep reagents (Applied Biosystems, USA) (Ferreira P E, unpublished data). In brief, for the third method, the biological samples were lysed in three-fold volume of NucPrep reagents. The lysate mixture was incubated for 1h (instead of 10 min) at 58°C, and the lysed samples were incubated at 4°C overnight before preforming the extraction (as previously reported) [7]. On day two, the solid material was separated from the lysate by passing the content through a 5 mL syringe. The full lysate was flowed through one column per sample in a DNA purification tray II (Applied Biosystems, USA) by three consecutive loadings. DNA washing was performed as recommended by the manufacturer. Incubation with DNA elution solution 1 was increased from three to five minutes. The final DNA containing elution volume was 200 μL for ABI extraction, 50 μL in the simple elution method and ~190 μL in the Chelex-100 extraction (after deduction of 5% Chelex-100 from a total volume of 200 μL).

### RDT devices

DNA extraction was compared from two RDT devices of clinical importance in Zanzibar: Paracheck-Pf® (Orchid Biomedical Systems, Goa, India) and SD-Bioline Malaria Ag P.f/Pan® (Standard Diagnostic, Inc, USA). Paracheck-Pf has been widely used in sub-Saharan Africa, and was the first RDT to be implemented in Zanzibar in 2006. Zanzibar has recently changed to SD-Bioline P.f/Pan as this test also detects species other than *P. falciparum*.

### *Plasmodium falciparum in vitro* samples and analysis

Both RDT devices were seeded according to the manufacturers’ instructions with 5 μL of 10-fold serial dilutions of laboratory cultured 3D7 *P. falciparum* (200,000-0.02 parasites/μL) as well as with malaria negative whole blood (negative control). For comparison, Whatman® 3MM filter paper was seeded in parallel with 5 μL (approximately equivalent to one 3-mm punch) of the serial dilution. Parasite cultures and malaria negative whole blood were lysed by freeze-thawing prior to serial dilution.

**Figure 1:**
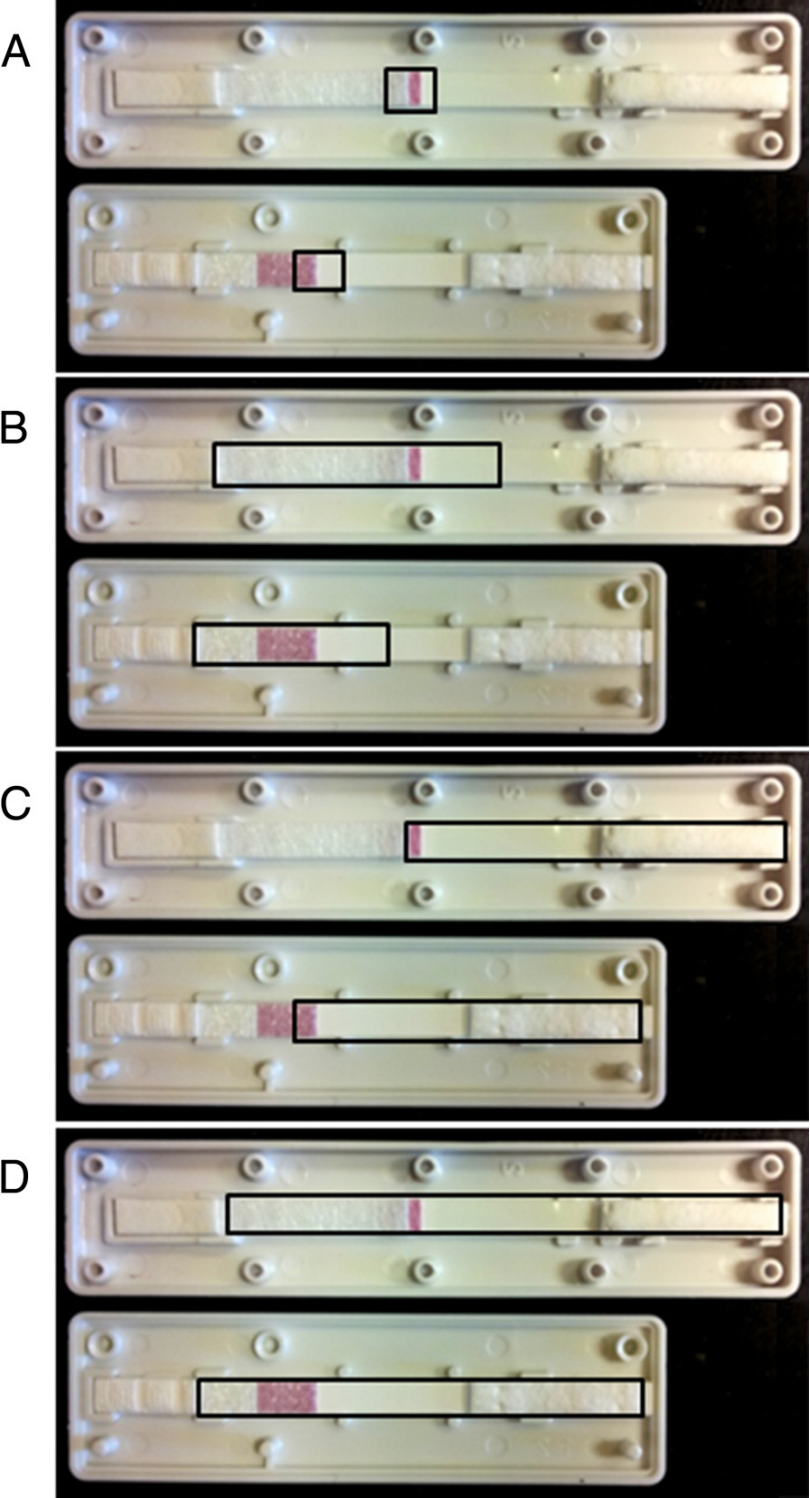
**RDT fragments used for DNA extraction.** Each panel shows Paracheck-Pf (top) and SD-Bioline Malaria Ag P.f/Pan (bottom). The RDTs are orientated with the buffer pad and sample site to the left (proximal end), and the absorption pad to the right (distal end). The fragments used for extraction are outlined by the black boxes **A**) 1cm **B**) Proximal **C**) Distal **D**) Whole.

The RDTs and filter papers were allowed to air dry for a minimum of 16 hours at room temperature (20°C), whereafter RDT cassettes were opened using a thin metal spatula. The nitrocellulose strip was held at the buffer pad with forceps and cut into 3 × 3 mm pieces using scissors. In between each sample, forceps and scissors were washed in 70% ethanol and dried on clean tissue paper, to minimise cross-contamination during sample preparation. RDT preparation was in accordance with the worldwide antimalarial resistance network (WWARN) guidelines, with minor modifications [8]. DNA extraction was compared from four different RDT fragments (Figure 1), all including the proximal third of the nitrocellulose strip that has been reported to generate best results upon DNA extraction [3]. DNA was extracted using the three methods described above.

The *P. falciparum* DNA detection limits were determined using three PCR techniques: 18S ribosomal DNA (rDNA) nested PCR [9], cytochrome b nested PCR [10], and 18S rDNA probe-based real-time PCR [11]. The same volume of DNA was used from each extraction method (2-5 μL depending on PCR). The *P. falciparum* detection limits were determined as the lowest consecutive positive sample in the dilution series.

### Field study sampling

The field samples were collected during an RDT effectiveness study conducted in 12 public health facilities, six each in North A and Micheweni districts, Zanzibar, May-July 2010 (Shakely *et al.*, submitted). Febrile patients were tested for *P. falciparum* malaria with Paracheck-Pf. In parallel approximately 100 μL of blood was spotted onto Whatman 3MM filter paper. Paired RDT and filter paper samples were available from 121 RDT positive and 734 RDT negative patients. Informed consent was obtained from enrolled patients or parent/guardians of children. The study was conducted in accordance with the Declaration of Helsinki [12] and Good Clinical Practice [13]**.** The study is registered as http://NCT01002066. Ethical approvals were obtained from the ethical committee in Zanzibar (ZAMREC/ST/0021/09) and the Regional Ethics Committee in Stockholm, Sweden (2009/387-31). All samples were stored at room temperature with desiccants until the time of DNA extraction. Samples were transported to Karolinska Institutet, Sweden, August 2010, where DNA extraction from RDT and filter paper samples was conducted in parallel within six months after sample collection.

### Field sample analysis

DNA was extracted from the paired RDT and filter paper field samples (N=855) using the ABI PRISM 6100 Nucleic Acid PrepStation™ method. DNA was extracted from the distal two thirds (Figure 1C) of the RDT strip, containing ~5 μL blood, as described above, and from three 3-mm punches of filter paper, containing ~15 μL blood, as described previously [7].

RDT negative samples (N=734) were screened using an 18S rDNA real-time PCR assay, that detects all five species of *Plasmodium*[14]. Samples were pooled two by two in 384 well plates. Each PCR was performed twice at different time points. Pools with a single Cycle threshold (Ct) value <40, or a Ct average <42 were selected for multiplex species identification for *P. falciparum*, *Plasmodium vivax*, *Plasmodium ovale* and *Plasmodium malariae*. Samples were considered PCR positive if positive in the species identification analysis.

Single nucleotide polymorphisms (SNPs) in *pfcrt* K76T, *pfmdr1* N86Y, Y184F and D1246Y were analysed with previously described PCR-RFLP methods in all RDT positive samples (N=121) [15-17]. An infection was defined as mixed when both alleles were present at a particular locus. *Pfmdr1* copy number was determined by the comparative ΔΔCt method following a TaqMan probe-based real-time PCR [18]. Samples were considered PCR positive if positive in at least one PCR.

All PCRs were run in parallel on RDT and filter paper extracted DNA.

### Statistical analysis

SNP genotyping outcomes were compared between RDT and filter paper extracted DNA by kappa analysis (κ). The SNP and haplotype prevalences were analysed and published elsewhere [17]. The spread of Ct values below the cut-off of Ct 35, in the TaqMan probe-based real-time PCR for determining *pfmdr1* copy number, were compared by Wilcoxon rank-sum test, as there were many incomplete pairs. All calculations were done with Stata/SE 12.0, StataCorp LP USA. Statistical significance was defined as p<0.05.

## Results

### *In vitro* cultured parasites - sensitivity of RDT-DNA extraction methods

The *P. falciparum* DNA detection limit varied with RDT device and extraction method (Table 1). When following the respective protocols, Chelex-100 extraction preformed best for both RDT devices as well as for extraction from filter paper, with a detection limit of two parasites/μL. The ABI extraction method had a 10-fold higher detection limit of 20 parasites/μL. DNA extraction was generally more efficient from SD-Bioline Malaria Ag P.f/Pan than from Paracheck-Pf. The simple elution method was unsuccessful for DNA extraction from Paracheck-Pf. Increasing the size of the nitrocellulose strip fragment (as seen in Figure 1) did not improve the level of detection. The method of *P. falciparum* detection influenced the detection limit by one to two log units (see Additional file 1). DNA extraction from RDTs was generally equal to or better than DNA extraction from an equal volume (5 μL) of blood spotted on filter paper.

**Table 1:** **Sensitivity of RDT-DNA extraction methods in *in vitro *cultured parasites**

**RDT Fragment**	**Simple elution**	**Chelex-100**	**ABI**
Paracheck-Pf			
1 cm	§	2	200
Proximal	NA	20	200
Distal	NA	NA	20
Whole	NA	NA	20
SD-Bioline Malaria P.f/Pan			
1 cm	2	2	20
Proximal	NA	2	20
Distal	NA	NA	20
Whole	NA	NA	20
Filter paper			
5 μL blood spot	200	2	200

Lowest achieved parasite detection levels (parasites/μL) for DNA extraction from Paracheck-Pf, SD-Bioline Malaria P.f/Pan and from 5 μL blood spotted on filter paper.

§ = Not estimated due to negative results.

NA = Not applicable, the size of the RDT fragment used for extraction was limited by the extraction volume.

### Field samples - parasite detection and drug resistance genotyping

There was no significant difference in PCR detection rates in DNA extracted from RDTs and filter paper. Out of 855 paired RDT and filter paper field samples, 118 (13.8%; CI 95% 11.4-16.2%) were PCR positive in both groups of samples (κ=0.94). Among the RDT negative field samples (N=734), three (0.4%; CI 95% 0.0-0.9%) and six (0.8%; CI 95% 0.1-1.5%) were PCR positive from RDT and filter paper extracted DNA, respectively (κ=0.44). Among the 121 RDT positive field samples, 115 (95.0%; CI 95% 91.1-99.0%) and 112 (92.6%; CI 95% 87.8-97.4%) were PCR positive (κ=0.50). No observed difference was found in the ability to detect low density parasitaemia (<100 parasites/μL), although the numbers were too small to allow for statistical analysis (12 PCR positives in both RDT and filter paper extracted DNA).

**Figure 2:**
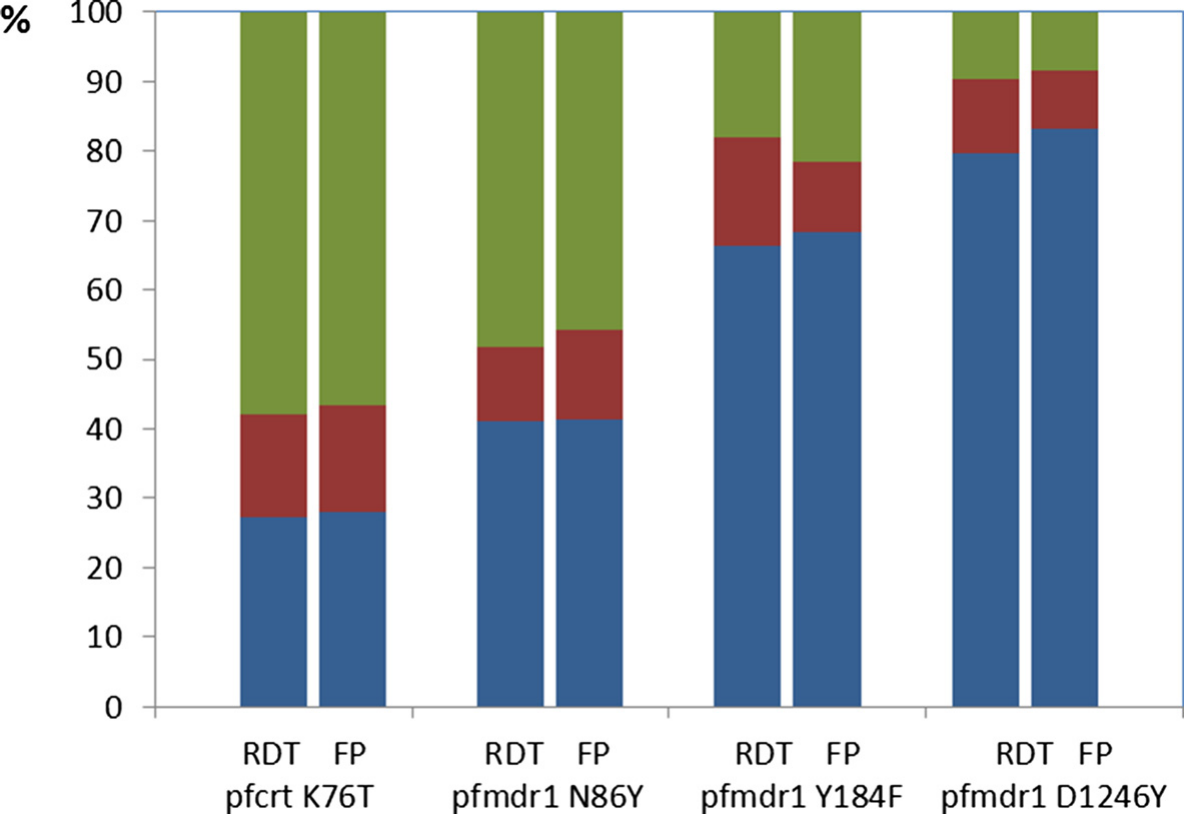
**Genotyping outcomes for RDT and filter paper extracted DNA at four key drug-resistance associated SNPs.** Stacked bar graph comparing the genotyping outcomes for RDT and filter paper extracted DNA for *pfcrt* K76T, *pfmdr1* N86Y, *pfmdr1* Y184F and *pfmdr1* D1246Y. Blue colour represents *pfcrt* K76, *pfmdr1* N86, Y184 and D1246, whereas red represents mixed infections, and green represents *pfcrt* 76T, *pfmdr1* 86Y, 184F and 1246Y, respectively.

There were no significant differences in PCR success rates and genotyping outcomes for the respective SNPs in the 121 RDT positive samples (Table 2). Furthermore, there was no difference between RDT and filter paper extracted DNA in the overall ability to detect mixed infections (Figure 2). Similarly, no statistically significant differences in the distribution of real-time PCR Ct values below Ct 35 from RDT and filter paper extracted DNA were observed (P_FAM_=0.10; P_VIC_=0.24). No sample contained multiple *pfmdr1* copy number.

**Table 2:** PCR success rates and agreement of genotyping outcomes in field samples

	**RDT PCR success rates N = 121 (%; CI 95%)**	**Filter paper PCR success rates N = 121 (%; CI 95%)**	**Kappa value**
*Pfcrt* K76T	114 (94.2; 89.9-98.5)	104 (86.0; 79.6-92.3)	0.72
*Pfmdr1* N86Y	112 (92.6; 87.8-97.4)	109 (90.1; 84.6-95.5)	0.85
*Pfmdr1* Y184F	110 (90.9; 85.7-96.2)	107 (88.4; 82.6-94.3)	0.74
*Pfmdr1* D1246Y	113 (93.4; 88.8-97.9)	107 (88.4; 82.6-94.3)	0.77
*Pfmdr1* copy number	84 (69.4; 61.0-77.8)	77 (63.6; 54.9-72.4)	-

Analyses of single nucleotide polymorphisms and *pfmdr1* gene copy numbers associated with anti-malarial drug resistance, from RDT and filter paper extracted DNA collected from 121 RDT positive field samples.

## Discussion

This is to date the most comprehensive comparative study of DNA extraction efficiency from malaria RDTs and assessment of the field applicability of RDT-DNA extraction for molecular surveillance, including detection of infections and key genetic markers associated with anti-malarial drug resistance.

DNA extraction efficiency from *in vitro* cultured *P. falciparum* varied with RDT device and extraction method. The same level of parasite detection as seen in previous publications was not achieved in this study [3,4]. Different designs of RDT devices affected the DNA extraction efficiency. In particular, DNA recovery from Paracheck-Pf was unsuccessful when employing the simple elution method, supporting as previously reported that plastic seals covering the nitrocellulose strip hamper DNA recovery [3].

The cost, time, final template volume and the purpose for DNA extraction should be considered when choosing extraction method. Although simple elution is the cheapest and fastest alternative, it is a crude method of DNA extraction and its use may be limited by RDT design and choice of PCR [3]. Chelex-100 is relatively inexpensive. The higher sensitivity observed with Chelex-100 extraction indicates that this method is particularly suitable for low density parasitaemia in low endemic settings. However, the Chelex-100 method is moderately labour-intensive and the DNA may be of lower quality than DNA extracted with commercially available column-based extraction kits [6]. Another concern is the storage capacity of Chelex-100 extracted DNA, which is thought to be more susceptible to DNA degradation during sample freeze-thawing [19]. Conversely, ABI extraction is a high throughput method providing high quality DNA, but at a substantial cost and requiring specialised equipment. This method had a higher *P. falciparum* detection level when compared with Chelex-100, perhaps explained by loss of DNA on the column. However, this did not appear to have influenced the field sample results (see below). Thus, ABI extraction could be suitable for analyses of RDT positive, symptomatic malaria patients enrolled in clinical trials. The final DNA containing volume is also important to take into consideration, as the concentration of the DNA will affect the parasite detection limits.

In the field analysis, RDTs provided DNA of equal quality as filter papers, suggesting that RDTs are a valuable alternative to filter paper for DNA storage in the field. High PCR success rates were obtained from DNA extracted from RDTs, for key loci in *pfcrt* and *pfmdr1* associated with anti-malarial drug resistance. SNP and haplotype prevalences were analysed and discussed elsewhere [17]. Wide scale collection of used RDTs is currently being implemented as an integral part of molecular surveillance of malaria in Zanzibar.

Increased deployment of RDTs in health care facilities and cross-sectional surveys facilitates passive and active collection of biological material for molecular surveillance. The advantages of using RDTs for DNA storage include reducing invasive procedures in the field. RDTs require just one finger prick for both malaria case detection and preservation of biological material. DNA storage on filter papers, on the other hand, requires an initial finger prick for malaria case detection by RDT or microscopy, followed by a second finger prick, for individuals with a positive diagnosis for collection of blood on filter paper. Multiple blood sampling can especially be problematic in small children and may increase the risk of mixing/miss-labelling of samples during collection. RDTs are also easily stored and have either a plastic or cardboard case that protects against cross-contamination. A disadvantage of RDT-DNA extraction is the limited amount of biological material (5–15 μL blood). This makes RDT-DNA extraction a “one shot operation” with no possibilities for re-extraction, unlike filter paper sampling where a larger amount of blood is usually collected (50–100 μL).

## Conclusions

The results support RDTs as a valuable source of parasite DNA and provide evidence for RDT-DNA extraction for improved malaria case detection, molecular drug resistance surveillance, and RDT quality control. However, the purpose of DNA extraction should be considered when choosing which extraction method best suits the type of samples to be analysed.

## Competing interests

The authors declare that they have no competing interests.

## Authors’ contributions

PF, UM, BA, AM, JPG and AB conceived and designed the study. DS, ASA and MIM carried out the field work. UM, BA and LJ carried out the molecular analyses. UM, BA, LJ, PF, AM and AB analysed the data. MP gave important intellectual input in the statistical analysis. UM drafted the manuscript. All authors read and approved the final manuscript.

## Supplementary Material

Additional file 1 **Sensitivity of RDT-DNA extraction methods in *in vitro* cultured parasites.** Description: Raw data showing detection levels for the three PCR methods used in the *in vitro* part of the study. Click here for file
