# Supplementary material for: Rapid diagnostic tests for molecular surveillance of Plasmodium falciparum malaria -assessment of DNA extraction methods and field applicability
Source: Malar J. 2013 Mar 19;12:106. doi: 10.1186/1475-2875-12-106 (PMC3605315; doi:10.1186/1475-2875-12-106)
Supplement: Additional file 1 — Sensitivity of RDT-DNA extraction methods in in vitro cultured parasites. Description: Raw data showing detection levels for the three PCR methods used in the in vitro part of the study. [file 1475-2875-12-106-S1.pdf]

Additional Data: Sensitivity of RDT-DNA extraction methods in *in vitro* cultured parasites. Raw Data showing detection levels for the three PCR methods used in the in vitro part of the study. A) 18S ribosomal DNA (rDNA) nested PCR, B) Cytochrome b nested PCR and C) 18s rDNA probe based real-time PCR. X marks a positive PCR in A and B; in C the minimum Cycle threshold (Ct) value is given. Detection limit was determined as the lowest consecutive positive.

**A) 18S rDNA nested PCR**			**Dilution series 1:10 (parasites /µL)**								
**Extraction Method**	**Extraction matrix**	**Extraction part**	**200,000**	**20,000**	**2000**	**200**	**20**	**2**	**0.2**	**0.02**	**0**
Simple elution	Paracheck-Pf	1cm	X								
	SD-Bioline	1cm	X	X	X	X	X				
	Filter paper	5µL	X	X	X						
Chelex-100	Paracheck-Pf	1cm	X	X	X	X					
		Proximal half	X	X	X	X					
	SD-Bioline	1cm	X	X	X	X	X				
		Proximal half	X	X	X	X					
	Filter paper	5µL	X	X	X	X	X				
ABI	Paracheck-Pf	1cm	X	X	X						
		Proximal half	X	X	X						
		Distal two-thirds	X	X	X						
		Whole RDT	X	X	X	X					
	SD-Bioline	1cm	X	X	X						
		Proximal half	X	X	X	X					
		Distal two-thirds	X	X	X						
		Whole RDT	X	X		X					
	Filter paper	5µL	X	X	X						

**B) Cytochrome b nested PCR**			**Dilution series 1:10 (parasites/µL)**								
**Extraction Method**	**Extraction matrix**	**Extraction part**	**200,000**	**20,000**	**2000**	**200**	**20**	**2**	**0.2**	**0.02**	**0**
Simple elution	Paracheck-Pf	1cm									
	SD-Bioline	1cm	X	X	X	X	X	X			
	Filter paper	5µL	X	X	X	X					
Chelex-100	Paracheck-Pf	1cm	X	X	X	X					
		Proximal half	X	X	X	X	X				
	SD-Bioline	1cm	X	X	X	X					
		Proximal half	X	X	X	X					
	Filter paper	5µL	X	X	X	X	X	X			
ABI	Paracheck-Pf	1cm	X	X	X	X					
		Proximal half	X	X	X	X					
		Distal two-thirds	X	X	X	X	X				
		Whole RDT	X	X	X	X					
	SD-Bioline	1cm	X	X	X	X	X				
		Proximal half	X	X	X	X		X			
		Distal two-thirds	X	X	X	X	X				
		Whole RDT	X	X	X	X	X				
	Filter paper	5µL	X	X	X	X					

C) 18s rDNA probe based real-time PCR			Dilution series (1:10 parasites/µL)								
**Extraction Method**	**Extraction matrix**	**Extraction part**	**200, 000**	**20, 000**	**2000**	**200**	**20**	**2**	**0.2**	**0.02**	**0**
Simple elution	Paracheck-Pf	1cm	NA	NA	NA	NA	NA	NA	NA	NA	NA
	SD-bioline	1cm	NA	NA	NA	NA	NA	NA	NA	NA	NA
	Filter paper	5µL	NA	NA	NA	NA	NA	NA	NA	NA	NA
Chelex-100	Paracheck-Pf	1cm	28.4	27.7	31.5	35.6	37.5	39.6			
		Proximal half	24.1	27.1	30.6	33.7	37.5				
	SD-Bioline	1cm	24.0	27.7	35.1	33.7	36.9	39.7			
		Proximal half	24.3	28.3	31.6	34.5	36.0	39.3			
	Filter paper	5µL	25.7	27.3	30.6	34.2	36.3	38.3			
ABI	Paracheck-Pf	1cm	27.4	33.5	35.6	40.5					
		Proximal half	26.4	30.2	34.6	37.2		39.4			
		Distal two-thirds	26.7	32.8	36.3	41.0					
		Whole RDT	28.3	31.8	34.3	37.3	40.3				
	SD-Bioline	1cm	29.3	33.9	37.3	40.0	40.1				
		Proximal half	29.2	32.0	36.7	38.6	40.1				
		Distal two-thirds	28.0	31.0	34.0	37.4					
		Whole RDT	29.5	32.9	36.1	38.8	39.0				
	Filter paper	5µL	32.8	36.3	39.1						

NA = Not determined due to the limiting DNA extraction volume.
